# Overexpression of enzymes in glycolysis and energy metabolic pathways to enhance coenzyme Q10 production in *Rhodobacter sphaeroides* VK-2-3

**DOI:** 10.3389/fmicb.2022.931470

**Published:** 2022-08-12

**Authors:** Long Zhang, Yong-li Li, Jian-hua Hu, Zhan-ying Liu

**Affiliations:** ^1^Inner Mongolia Energy Conservation and Emission Reduction Engineering Technology Research Center for Fermentation Industry, Hohhot, Inner Mongolia, China; ^2^Engineering Research Center of Inner Mongolia for Green Manufacturing in Bio-fermentation Industry, Hohhot, Inner Mongolia, China; ^3^College of Chemical Engineering, Inner Mongolia University of Technology, Hohhot, Inner Mongolia, China

**Keywords:** *Rhodobacter sphaeroides*, metabolic engineering, coenzyme Q10, glycolysis metabolic pathways, energy metabolic pathways

## Abstract

We subjected the components of the glycolysis and energy metabolism pathways of *Rhodobacter sphaeroides* (*R*. *sphaeroides*) to metabolic engineering to improve the titer and yield of coenzyme Q10 (CoQ10). Phosphofructokinase (PFK), cyclic adenylate-dependent protein kinase (PKAC), glyceraldehyde-3-phosphate dehydrogenase (GAPDH), and adenosine triphosphate hydrolase (KdpC) were overexpressed in *R*. *sphaeroides* VK-2-3 (VK-2-3). The strains were labeled *R*. *sphaeroides* PFK (RS.PFK), RS.PKAC, RS.PFK–PKAC, RS.KdpC, RS.GAPDH, and RS.KdpC–GAPDH. Results showed that the CoQ10 titers of RS.PFK, RS.PKAC, and RS.PFK–PKAC were 300.96 ± 0.87, 405.94 ± 4.77, and 379.94 ± 0.42 mg/l, respectively. The CoQ10 titers of RS.PFK and VK-2-3 were not significantly different; however, those for RS.PKAC and RS.PFK–PKAC were 13 and 6% higher than that of VK-2-3, respectively. Further, the titers of RS.KdpC, RS.GAPDH, and RS.KdpC–GAPDH were 360.17 ± 0.39, 409.79 ± 0.76, and 359.87 ± 1.14 mg/l, respectively. The titers of RS.KdpC and RS.KdpC–GAPDH were not significantly different from that for VK-2-3, whereas that for RS.GAPDH was 14% higher than that of VK-2-3. Finally, when the cultures of RS.GAPDH and VK-2-3 were scaled up in 5-L fermenters, the CoQ10 titers and RS.GAPDH yields increased by 44.3 and 37.8%, respectively, compared with VK-2-3.To the best of our knowledge, the glycolysis pathway of *R. sphaeroides* was studied for the first time in this study. We genetically modified the components of the energy metabolism pathway to obtain the strain with high yield of CoQ10 mutant RS.GAPDH. The findings of this study can serve as a basis for future studies involving metabolic engineering of CoQ10-producing strains.

## Introduction

Coenzyme Q10 (CoQ10) acts as an electron transporter in the respiratory chain (Cluis et al., 2012) and participates in cellular biochemical reactions. CoQ10 is widely used in cosmetics, food additives, and health products (Littarru and Tiano, 2010; Brandmeyer et al., 2014; Campagnolo et al., 2017; Hernández-Camacho et al., 2018). The primary method of CoQ10 production is microbial fermentation by bacterial strains such as *Rhodopseudomonas copsulata* (Meganathan, 2001), *Agrobacterium tumefaciens* (Choi et al., 2005), and *Rhodobacter sphaeroides* (*R. sphaeroides*; Yoshida et al., 1998). *R. sphaeroides* is widely used in CoQ10 production. Ya (2010), Chen (2016), and Qian (2016) have previously applied physical and chemical mutagenesis in *R. sphaeroides* to improve the titers and yield of CoQ10. Wang et al. (2021) attempted iterative mutagenesis through room-temperature plasma treatment to increase the production of CoQ10 in *R. sphaeroides*. After two rounds of mutagenesis screening, a mutant strain, RS 17, exhibited an 80.37% increase in CoQ10 titers compared with the control. Zou et al. (2019) used atmospheric and room temperature plasma for mutagenesis of *R. sphaeroides* combined with vitamin K3 resistance screening to obtain a mutant, AR01. After 100 h of fermentation, the CoQ10 titer of AR01 was 590 mg/l, which is 25.5% higher than that of the parental strain. However, random mutation breeding has the disadvantages of requiring heavy labor, being tedious, and introducing unwanted genetic changes.

With the rapid development of molecular biology techniques and in-depth understanding of the CoQ10 synthetic pathway, rational genetic engineering was applied to induce targeted regulation (Davies and Brindle, 1992; Kolkman and Stemmer, 2001; Nielsen and Olsson, 2002; Liu et al., 2013; Lv et al., 2014) and modification (Scalcinati et al., 2012; Lu et al., 2014, 2015; Zhu et al., 2017; Tsuge et al., 2019) in cells to precisely regulate the metabolic pathway and obtain strains producing high-yield target products. For instance, Zhu (2017) induced the overexpression of glyceraldehyde-3-phosphate dehydrogenase (GAPDH) and inhibited carotenoid synthesis pathway to achieve CoQ10 production in *R. sphaeroides* of up to 63.7 mg/l, which was 27.8% higher than the control. Based on the literature, modifications in CoQ10 production by *R. sphaeroides* focus on quinone ring synthesis, quinone ring modification, and side-chain synthesis pathways (Lu et al., 2014, 2015).

**Figure 1:**
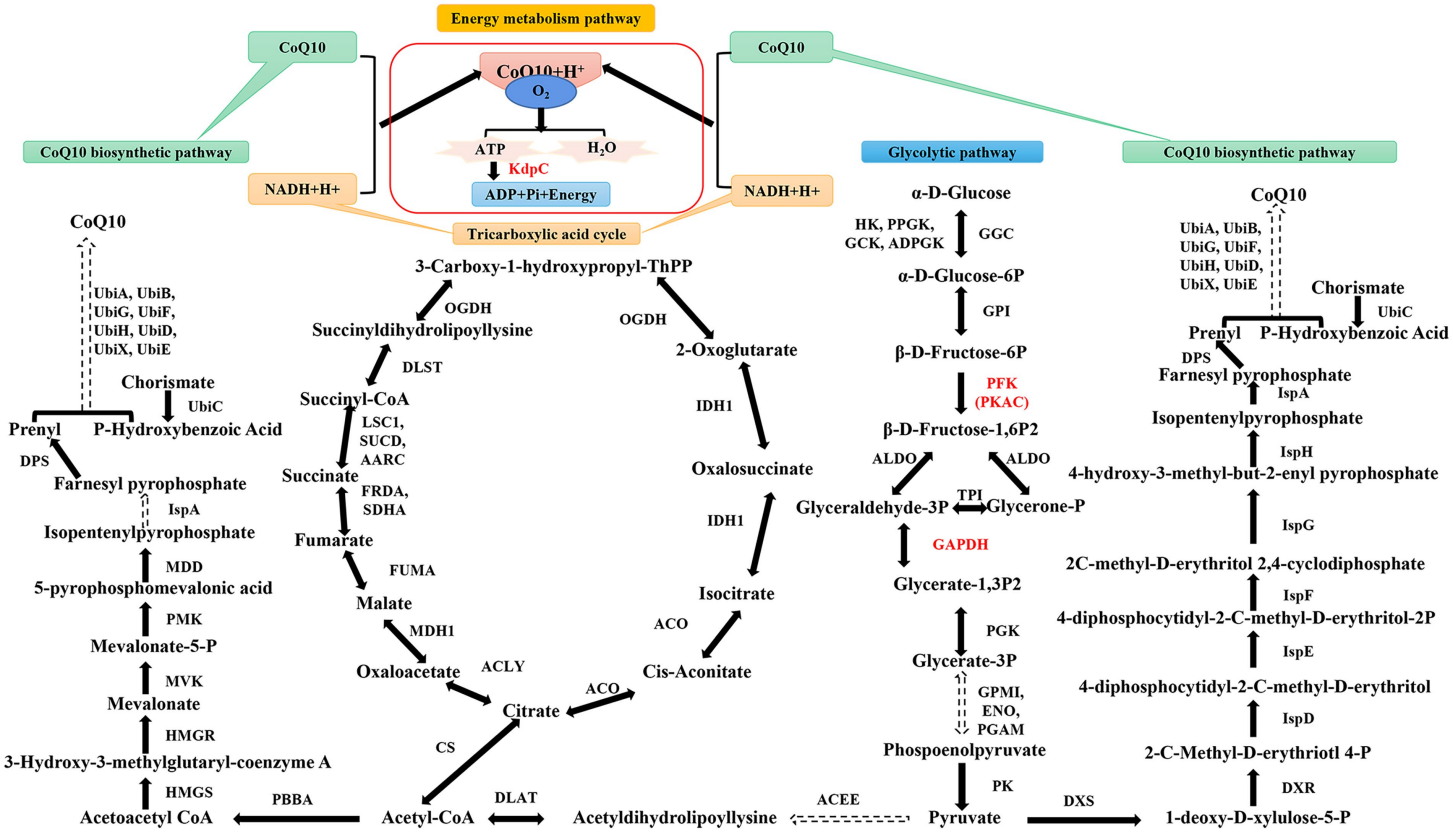
Simplified diagram of glycolysis, tricarboxylic acid cycle, and the coenzyme Q10 (CoQ10) synthesis pathway. HK, hexokinase; PPGK, polyphosphate glucokinase; GCK, glucokinase; ADPGK, ADP-dependent glucokinase; P, phosphate; GPI, glucose-6-phosphate isomerase; ALDO, fructose-bisphosphate aldolase; PGK, phosphoglycerate kinase; PGAM, 2,3-bisphosphoglycerate-dependent phosphoglycerate mutase; GPMI, 2,3-bisphosphoglycerate-independent phosphoglycerate mutase; ENO, enolase; PK, pyruvate kinase; ACEE, pyruvate dehydrogenase E1 component; DLAT, pyruvate dehydrogenase E2 component; CS, citrate synthase; ACLY, ATP citrate; MDH1, malate dehydrogenase; FUMA, fumarate hydratase; FRDA, fumarate reductase flavoprotein subunit; SDHA, succinate dehydrogenase flavoprotein subunit; LSC1, succinyl-CoA synthetase alpha subunit; SUCD, succinyl-CoA synthetase alpha subunit; AARC, succinyl-CoA: acetate CoA-transferase; DLST, 2-oxoglutarate dehydrogenase E2 component; OGDH, 2-oxoglutarate dehydrogenase E1 component; IDH1, isocitrate dehydrogenase; ACO, aconitate hydratase; PBBA, acetoactyl-CoA thiolase; HMGS, 3-Hydroxy-3-methylglutaryl-coenzyme A synthetase; HMGR, 3-Hydroxy-3-methylglutaryl-coenzyme A reductase; MVK, mevalonate kinase; PMK, phosphomevalonate kinase; MDD, mevalonate 5-pyrophosphate decarboxylase; IspA, farnesyl diphosphate synthase; DPS, isoprenyl diphpsphate synthase; UbiC, chorismate lyase; UbiA, 4-hydroxybenzoate polyprenyltransferase; UbiB, ubiquinone biosynthesis protein; UbiG, 3-demethylubiquinone-9 3-methyltransferase; UbiF, 3-demethoxyubiquinol 3-hydroxylase; UbiH, 2-octaprenyl-6-methoxyphenol hydroxylase; UbiD, 4-hydroxy-3-polyprenylbenzoate decarboxylase; UbiX, 3-octaprenyl-4-hydroxybenzoate carboxy-lyase; UbiE, demethylmenaquinone methyltransferase; DXS, 1-deoxy-D-xylulose-5-phosphate synthase; DXR, 1-deoxy-D-xylulose-5-phosphate reductoisomerase; IspD, 2-C-methyl-D-erythritol 4-phosphate cytidylyltransferase; IspE, 4-diphosphocytidyl-2-C-methyl-D-erythritol kinase; IspF, 2-C-methyl-D-erythritol 2,4-cyclodiphosphate synthase; IspG, (E)-4-hydroxy-3-methylbut-2-enyl-diphosphate synthase; IspH, 4-hydroxy-3-methylbut-2-enyl diphosphate reductase. PFK, PKAC, KdpC, GAPDH, and CoQ10 are the enzymes and metabolites of interest in this study. Solid lines indicate one-step reactions and dashed lines indicate the intermediate multi-step reactions. Two-way arrows represent reversible reactions.

The precursor for CoQ10 synthesis is derived from glycolysis, and phosphofructokinase (PFK) is the rate-limiting enzyme in the pathway. In addition, cyclic adenylate-dependent protein kinase (PKAC) can be used as a cofactor for CoQ10 synthesis (Figure 1). Moreover, as an electron transporter in the respiratory chain, CoQ10 is crucial for energy conversion. GAPDH and adenosine triphosphate hydrolase (KdpC) are associated with energy conversion; however, the mechanisms of these components have rarely been investigated. Therefore, in this study, the essential genes of the CoQ10 synthesis pathway in *R. sphaeroides* were subjected to metabolic regulation regarding glycolysis and energy conversion to enhance CoQ10 production. Finally, the engineered strain producing strains with high yield of CoQ10 was expanded in a bioreactor. This study aimed to provide a novel method for applying metabolic engineering to CoQ10 production.

## Materials and methods

### Strains and culture media

*Escherichia coli* DH5α containing pBBR1MCS-4 plasmid was cultivated at 37°C in Luria–Bertani medium (tryptone 10 g/l, yeast extract 5 g/l, and NaCl 10 g/l) supplemented with 100 μg/ml of ampicillin. *R*. *sphaeroides* VK-2-3 (VK-2-3, CCTCC M 2021735) was obtained by subjecting the original strain of *R. sphaeroides* (V-0) to compound mutagenesis using heavy ions and high-voltage prick electric fields. Information on strains and plasmid sources was described in detail in the Supplementary Table S4. VK-2-3 and its recombinant derivatives were cultivated in medium A containing 3 g/l glucose, 8 g/l yeast extract powder, 2 g/l NaCl, 1.3 g/l KH_2_PO_4_, 0.25 g/l MgSO_4_·7H_2_O, 20 g/l agar powder, and 1 ml/l auxiliary solution, and the pH of the medium was adjusted to 7.16 using 10% NaOH. Medium B was used for the shake-flask seed culture and contained 3 g/l glucose, 8 g/l yeast extract powder, 2 g/l NaCl, 1.3 g/l KH_2_PO_4_, 0.25 g/l MgSO_4_·7H_2_O, 0.01 g/l CoCl_2_·6H_2_O, and 1 ml/l auxiliary solution; the pH of this medium was adjusted to 7.0 using 10% NaOH. Medium C was used for the shake-flask fermentation culture and contained 35 g/l glucose, 12 g/l corn syrup powder, 3 g/l C_5_H_8_NO_4_Na, 3 g/l NaCl, 3 g/l (NH_4_)_2_SO_4_, 3 g/l KH_2_PO_4_, 12.5 g/l MgSO_4_·7H_2_O, 12 g/l CaCO_3_, 0.01 g/l CoCl_2_·6H_2_O, and 1 ml/l auxiliary solution; the pH of this medium was adjusted to 7.0 using 10% NaOH.

The basic medium of the fermentation tank comprised 25 g/l glucose, 6.5 g/l C_5_H_8_NO_4_Na, 4 g/l corn steep liquor, 4 g/l (NH_4_)_2_SO_4_, 4 g/l NaCl, 0.03 g/l MnSO_4_, 1.2 g/l KH_2_PO_4_, 0.01 g/l CoCl_2_·6H_2_O, 8 g/l MgSO_4_·7H_2_O, 0.08 g/l NaBO_3_·4H_2_O, 1.2 g/l FeSO_4_·7H_2_O, 0.05 g/l CaCl_2_, and 8 g/l CaCO_3_; the pH was adjusted to 6.5 using NH_3_·H_2_O. The supplemental medium of the fermentation tank comprised 6.5 g/l C_5_H_8_NO_4_Na, 4 g/l dry corn steep powder, 4 g/l (NH_4_)_2_SO_4_, 4 g/l NaCl, 0.03 g/l MnSO_4_, 0.01 g/l CoCl_2_·6H_2_O, 8 g/l MgSO_4_·7H_2_O, 1.2 g/l FeSO_4_·7H_2_O, 0.08 g/l NaBO_3_·4H_2_O, and 0.05 g/l CaCl_2_. The flow feeding for the fermentation tank was prepared using 2 l of 60% glucose solution, 150 ml of 7% KH_2_PO_4_ solution, 400 ml of NH_3_·H_2_O at a concentration of 25% for adjusting pH, and 40 ml of defoamer.

### Target fragment amplification

Routine methods of molecular cloning, such as PCR, deoxyribonucleic acid restriction digestion, and ligation, were conducted in accordance with standard protocols. Whole genomes of V-0 (PRJNA818168) and VK-2-3 (PRJNA818446) were sequenced, and genes encoding PFK, PKAC, GAPDH, and KdpC were annotated using the online annotation service of the Kyoto Encyclopedia of Genes and Genomes Orthology database.^1^ PCR was performed using the VK-2-3 genome as a template to obtain the target fragment. The target fragment was recovered using an agarose gel recovery kit.

### Construction of recombinant strains

The target fragment and pBBR1MCS-4 were double digested using *Bam*HI, *Hin*dIII, and *Kpn*I and ligated using T4 DNA ligase. The recombinant plasmids were transferred into *E. coli* DH5α culture, plated on ampicillin-coated (100 μg/ml) plates, and incubated at 37°C overnight. The recombinant plasmids were sequenced at Shanghai Biotech. The plasmids were transformed into VK-2-3 *via* electroporation for strain construction. Recombinant plasmid construction and other detailed methods are presented in Supplementary Figure S1. The sequencing results for *pfk*, *pkac*, *kdpc*, and *gapdh* correspond with sequences 1 (ON381190), 2 (ON381191), 3 (ON381192), and 4 (ON381193) in Supplementary material, respectively.

### Setting fermenter parameters

The seed solution (250 ml) was inoculated into a 5-L fermenter at a 2.5-L loading volume. The fermentation temperature was set to 32°C, and the pH was adjusted to 6.7 using 25% NH_3_·H_2_O. The stirring paddle speed was set to 500 rpm, and the ventilation ratio was 1.8 vvm. Glucose concentration was controlled at 0.8–1.2% in the fermentation broth. A sugar-replenishing peristaltic pump replenished glucose every 50 s for 1 s. Glucose concentration in the fermentation broth was monitored online and manually corrected as required. The phosphate concentration in the fermentation broth was maintained at 80–120 ppm, and a phosphorus-replenishment peristaltic pump was used to replace phosphorus every 300 s for 1 s. Based on the readings, the phosphate concentration in the fermentation broth was monitored online and manually corrected as required. After the fermentation broth increased to 3.0 l, 500 ml of fermentation solution was collected from the pick-up port.

### Real-time quantitative PCR analysis

RNA was extracted from the fermentation broth using the Bacteria Total RNA Isolation Kit [Sangon Biotech (Shanghai) Co., Ltd.]. Agarose gel electrophoresis and the ultramicro nucleic acid protein detector EVA3100 (Monad Biotech Co., Ltd.) were used to determine RNA quality and concentration, respectively. This RNA was used as a template and reverse transcribed to cDNA using the SYBR® Premix Ex Taq II (Takara) kit; this procedure was performed on ice. The primer sequences for the genes were synthesized by Sangon Biotech (Shanghai) Co., Ltd. (Supplementary material; Supplementary Table S1). Real-time quantitative PCR (RT-qPCR) was performed using the TB Green Premix Ex Taq II kit (Takara) with the reverse-transcribed cDNA as a template. The entire procedure was performed on ice and away from light. The following reaction system was used: In a 384-well plate, 9 μl of the premix was added to each well (5 μl of TB Green Premix Ex Taq II, 1 μl each of forward and reverse primers, 1 μl of ROX Reference Dye II, 1 μl of RNA-free water). Finally, 1 μl of cDNA was diluted to 500 ng/μL and added to the wells. Gene expression was measured using a 384-well QuantStudio 6 Flex real-time fluorescence PCR assay system. The PCR reaction system was as follows: 95°C for 30 s, followed by 40 cycles of 95°C for 3 s, 60°C for 30 s, and 72°C for 20 s in 10 μl. At the end of the experiment, the melting curve was analyzed using the default parameters and *rpoZ* was used for normalisation (Zhu, 2017). All analyses were repeated three times using biological replicates.

### Analytical methods

#### Determining the growth curve

Absorbance values were measured at 600 nm from 0 to 30 h *via* spectrophotometry. The culture medium was diluted using sterile media to obtain an optical density (OD_600_) of 0.1–1.0. Finally, the true OD of the culture medium was multiplied by the dilution factor.

#### Determining residual glucose in the fermentation broth

The fermentation broth was centrifuged at 4200 rpm for 15 min. High-performance liquid chromatography (HPLC) was used to quantify residual glucose in the culture supernatants using the 1,290 Infinity System (Agilent Technologies, Waldbronn, Germany) equipped with the Aminex HPX-87H column (300 mm × 7.8 mm × 9 μm) and a refractive-index detector. The mobile phase comprised 5 mmol/l H_2_SO_4_ at a flow rate of 0.5 ml/min.

#### Determining the concentrations of NADH, NAD+, and ATP

The fermentation broth (5 ml) was centrifuged at 4°C and 8,000 rpm for 10 min. The obtained cell pellet was resuspended in 5 ml of 0.2 mol/l phosphate-buffered saline (pH = 7.0) and ultrasonically homogenized in an ice bath. Cell debris was removed by centrifugation and filtration. NADH, NAD^+^, and ATP were quantified using an HPLC system equipped with a C_18_ column (4.6 mm × 150 mm × 5 μm) and a UV detector at 254 nm. Specific details have been provided in the Supplementary Table S3, Supplementary Figure S2.

#### Determination of dry cell weight

Dry centrifuge tubes were weighed, and the mass was recorded as m_1_. The fermentation broth was centrifuged at 13,000 rpm for 10 min. The supernatant was removed and the tube was dried at 95°C until a constant weight was achieved; this mass was recorded as m_2_. Dry cell weight was calculated using the following formula: Dry cell weight = m_2_ − m_1_.

#### Determining CoQ10 concentration

CoQ10 was quantified using an HPLC system equipped with a C_18_ column (4.6 mm × 150 mm × 5 μm) and a UV detector at 275 nm. Methanol/ethanol (65: 35, *v/v*) was used as the mobile phase at 35°C and a flow rate of 1.0 ml/min. Quantification was conducted by calculating the peak area using the standard external method. All operations were performed in the dark.

##### Calculating CoQ10 yield and production

CoQ10 yield was calculated using Formula 1.


  (1) $$\mathrm{CoQ10}\;\mathrm{yield}\left(\mathrm{mg/g}\right)=\frac{\mathrm{CoQ10}\;\mathrm{titer}\;\left(\mathrm{mg/L}\right)}{\mathrm{Dry}\;\mathrm{cell}\;\mathrm{weight}\;\left(\mathrm{g/L}\right)}$$

CoQ10 production performance was calculated using Formula 2.


(2) $$\mathrm{Production performance}\;\left[\mathrm{mg}/\left(g\cdot g\cdot L\cdot h\right)\right]=\frac{A}{B\cdot C\cdot D\cdot E}$$

where A refers to CoQ10 titer; B refers to dry cell weight, C refers to sugar consumption, D refers to fermentation broth volume and E refers to fermentation time.

#### Data analysis

The experiments were performed using three biological replicates and data are expressed as means ± error bars. Origin 2021 was used for plotting, and Statistical Analysis System 9.2 was used to evaluate significant statistical differences. Relative expressions were calculated using the 2^−∆∆Ct^ method.

## Results and discussion

### Effects of overexpression of PFK and PKAC on CoQ10 production

**Figure 2:**
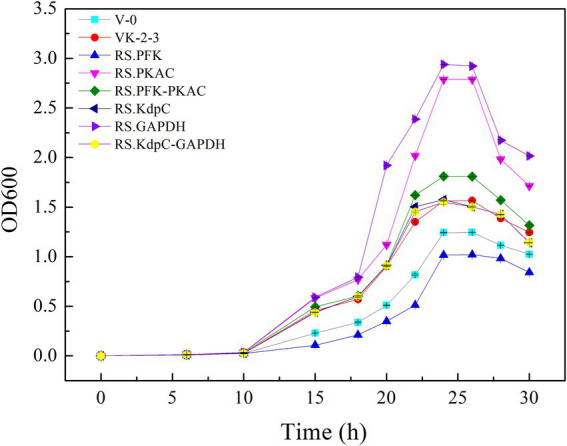
Growth curves of V-0, VK-2-3, and recombinant strains.

**Figure 3:**
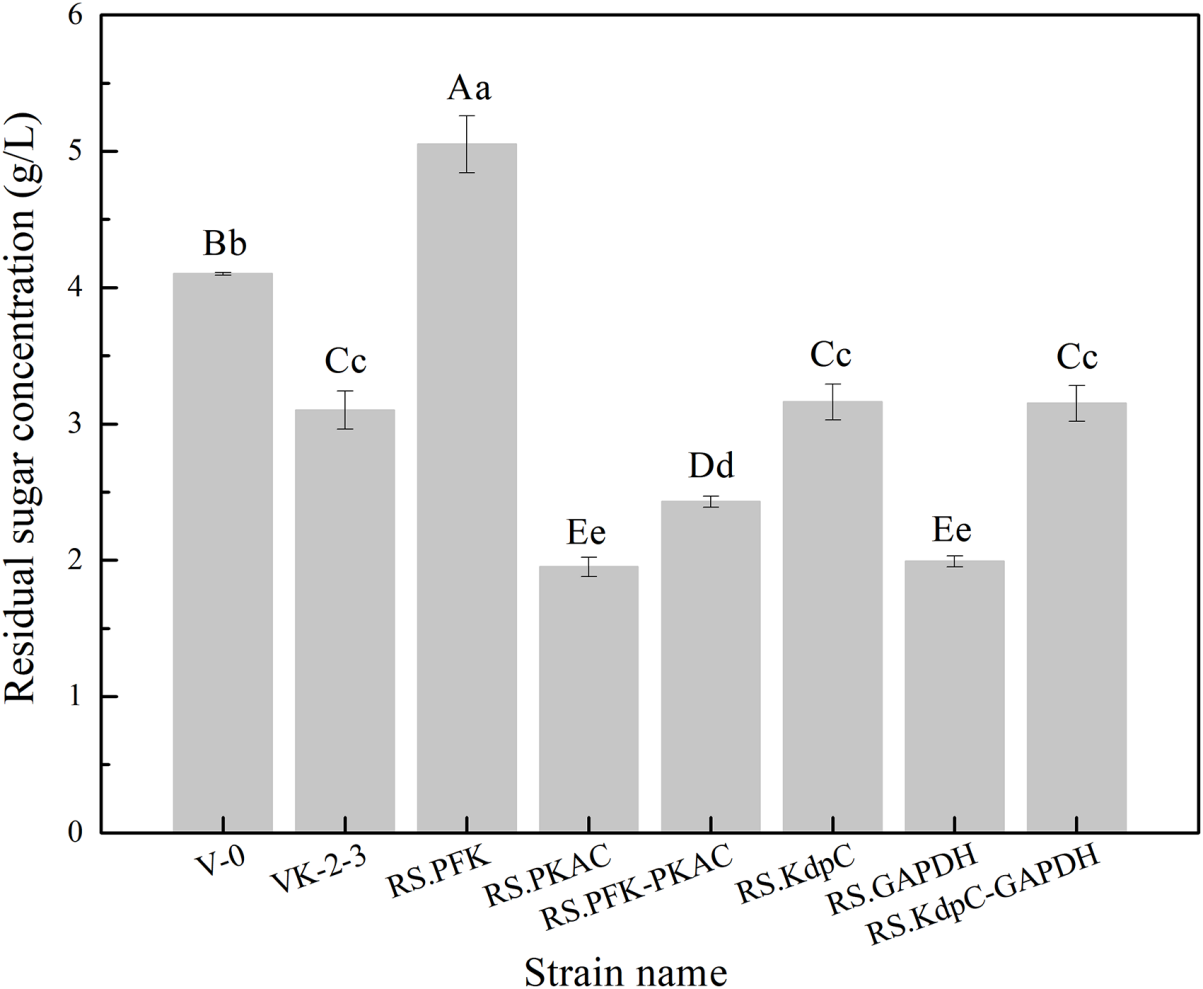
Residual sugar concentration in the fermentation broths of V-0, VK-2-3, and recombinant strains. Shoulder-notes in uppercase letters indicate highly significant differences (*p* < 0.01) and lowercase letters indicate significant differences (*p* < 0.05).

**Figure 4:**
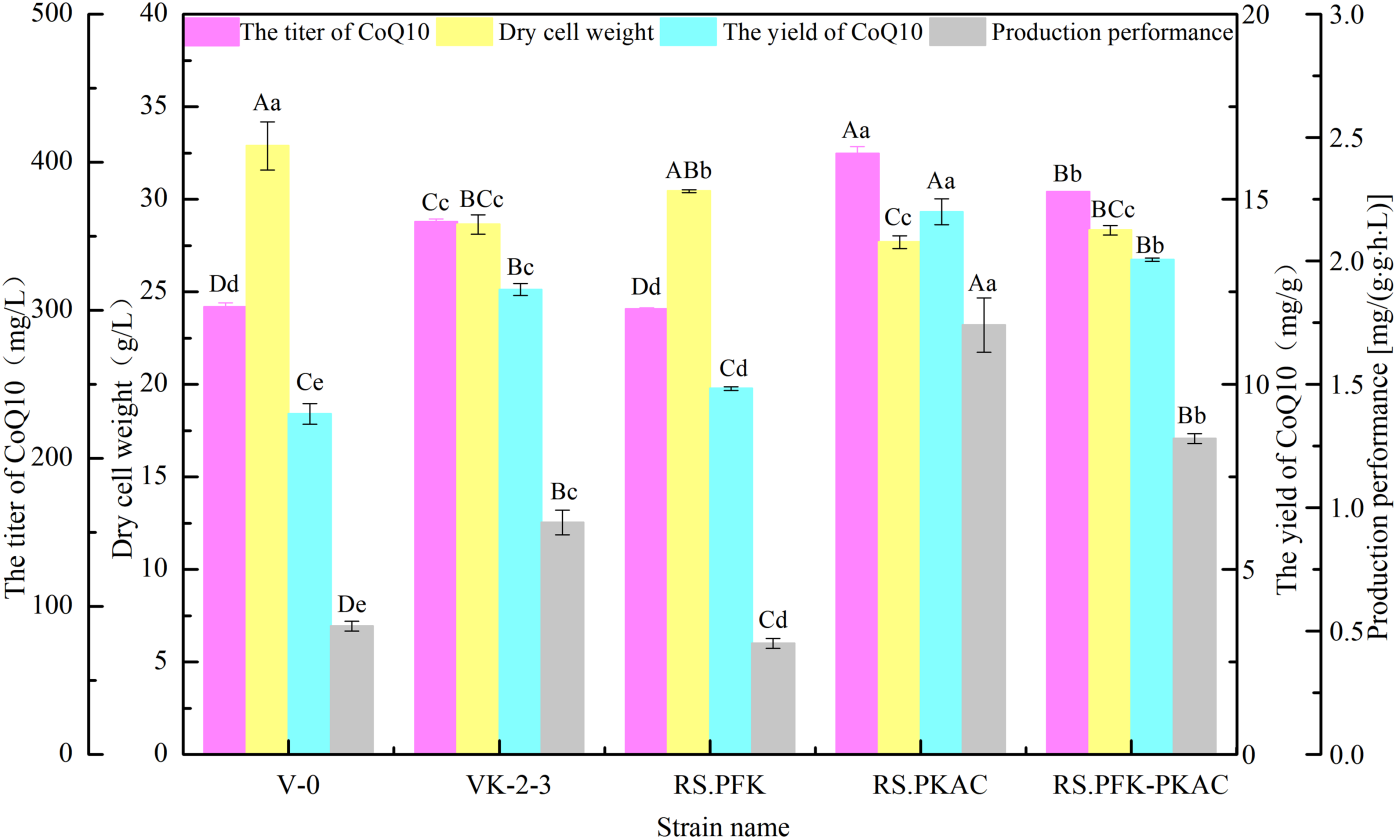
Fermentation test results of V-0, VK-2-3, RS.PFK, RS.PKAC, and RS.PFK–PKAC. Shoulder-notes in uppercase letters indicate highly significant differences (*p* < 0.01) and lowercase letters indicate significant differences (*p* < 0.05).

**Figure 5:**
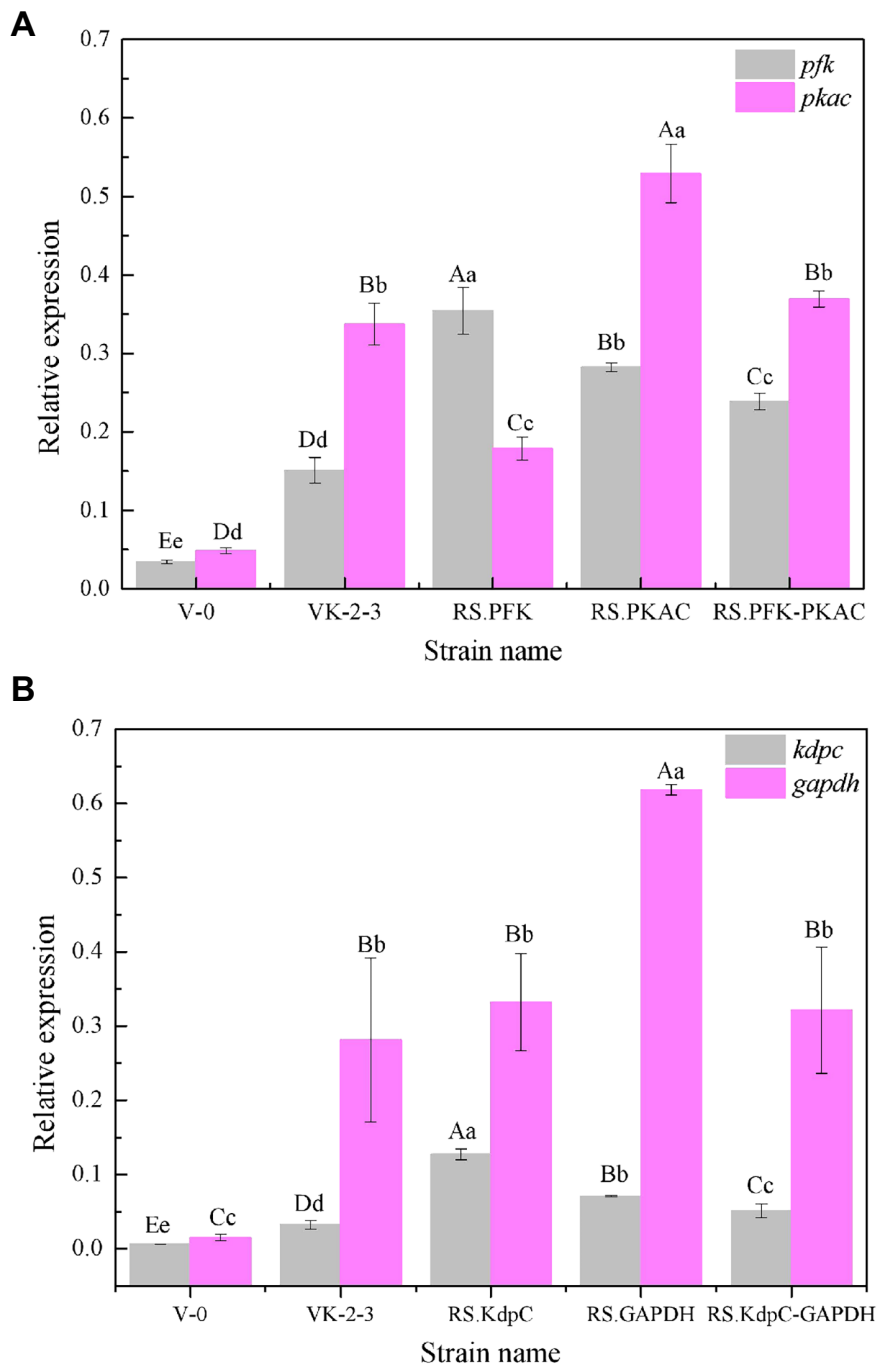
Relative expression of *pfk*, *pkac*, *kdpc*, and *gapdh* of V-0, VK-2-3, and recombinant bacteria. **(A)** Relative expression of *pfk* and *pkac* of V-0, VK-2-3, RS.PFK, RS.PKAC, and RS.PFK–PKAC. **(B)** Relative expression of *kdpc* and *gapdh* of V-0, VK-2-3, RS.KdpC, RS.GAPDH, and RS.KdpC–GAPDH. Shoulder-notes in uppercase letters indicate highly significant differences (*p* < 0.01) and lowercase letters indicate significant differences (*p* < 0.05).

The strains were in the lag phase within 0–10 h, began to grow after 10 h, and reached the stationary phase after 24 h. Compared with VK-2-3, RS.PKAC and RS.PFK–PKAC showed faster growth (Figure 2; Supplementary Table S2). Further, the residual sugar concentrations in the RS.PKAC and RS.PFK–PKAC fermentation broths were significantly lower than that in the VK-2-3 broth, whereas the residual sugar concentration in the RS.PFK fermentation broth was significantly higher than in the VK-2-3 (Figure 3; Supplementary Table S2). The CoQ10 titers of RS.PKAC and RS.PFK–PKAC significantly increased by 13 and 6%, respectively, compared with VK-2-3. The CoQ10 yield of RS.PKAC and RS.PFK–PKAC increased by 16.7 and 6.4% compared with VK-2-3, respectively (Figure 4; Supplementary Table S2). The RT-qPCR results revealed that the relative expression of *pfk* and *pkac* genes in the RS.PKAC strain was 1.9 and 1.6 times higher than those in VK-2-3, respectively. However, the relative expression of *pkac* gene decreased by 47% in RS.PFK strain compared with that in VK-2-3 (Figure 5A; Supplementary Table S2). Overexpression of PFK and PKAC to enhance target product production has rarely been reported in *R. sphaeroides*. Zhu et al. overexpressed PFK and PKAC in *Lactococcus lactis* (*L. lactis*). After 10 h of fermentation, The nisin yield of recombinant strain *L. lactis* N8-pMG36e-PFK–PKAC was increased by 20% compared with the wildtype strain (Zhu et al., 2015). Zhu et al. integrated additional copies of 1-deoxy-D-xylulose-5-phosphate synthase (*dxs*) and 1-deoxy-D-xylulose-5-phosphate reductoisomerase (*dxr*) into the *R. sphaeroides* genome, and the titer and yield of CoQ10 increased by 18.44 and 18.87%, respectively (Zhu et al., 2022). In this study, overexpression of *pfk* and *pkac* increased the growth and sugar consumption rates of RS.PKAC and RS.PFK–PKAC. The CoQ10 titer of RS.PKAC significantly increased compared with that of VK-2-3, indicating that the overexpression of a single gene can improve the efficiency of glucose utilization and increase cellular metabolism in *R. sphaeroides*. This metabolic flow pressure may be within the tolerance of the host cell, with little effect on its normal growth. However, the increase in the CoQ10 titer of RS.PFK–PKAC was less than that in the titer of RS.PKAC; this may be because two genes were coexpressed in tandem and the same promoter drove them. This further increased the metabolic load on the host cell beyond its tolerance and had a detrimental effect on its growth (Kim and Kim, 2004).

### Effects of overexpression of Kdpc and GAPDH on CoQ10 production

**Figure 6:**
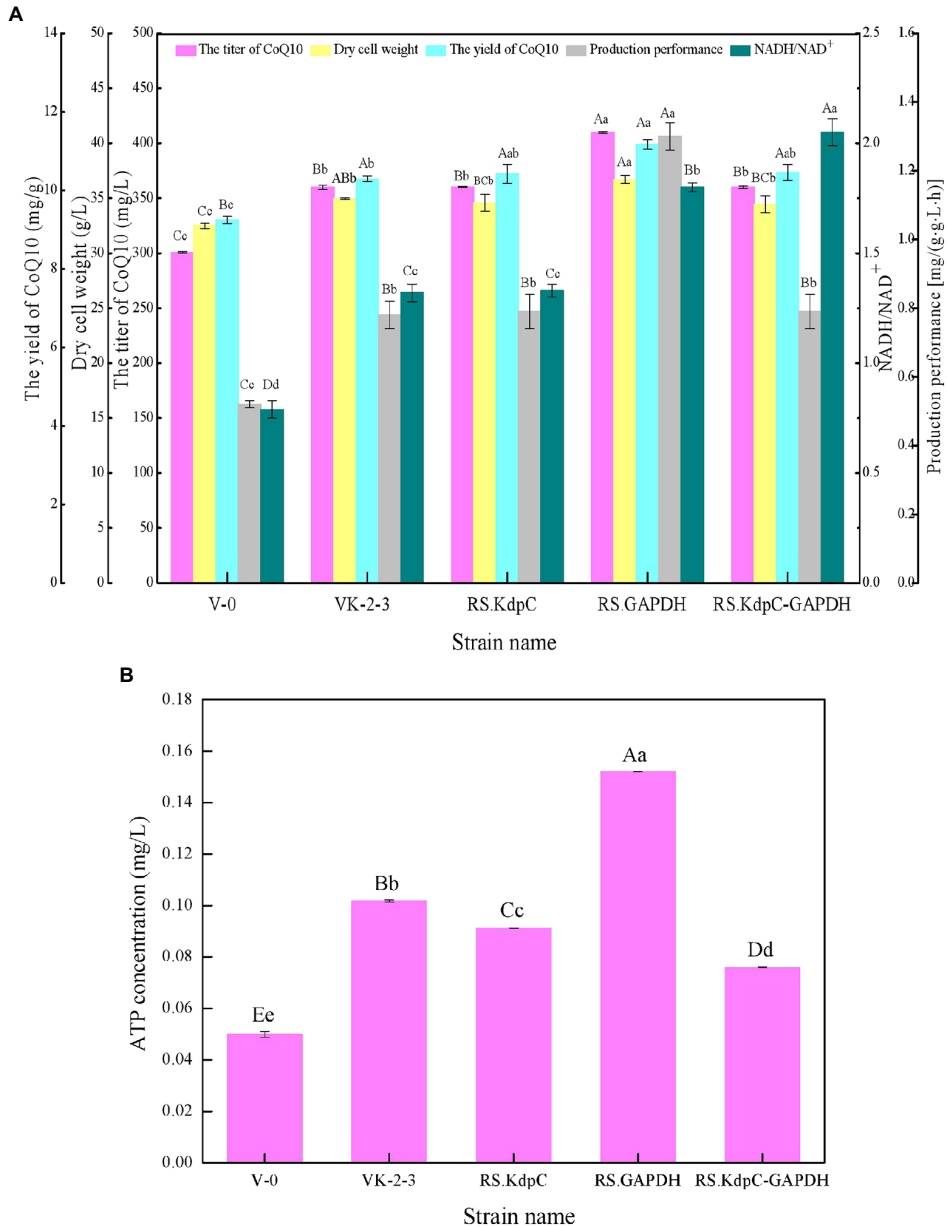
Fermentation test results of V-0, VK-2-3, RS.KdpC, RS.GAPDH, and RS.KdpC–GAPDH. **(A)** Coenzyme Q10 (CoQ10) titer, CoQ yield, dry cell weight, production performance, and NADH/NAD^+^. **(B)** ATP concentration. Shoulder-notes in uppercase letters indicate highly significant differences (*p* < 0.01) and lowercase letters indicate significant differences (*p* < 0.05).

The results showed that RS.GAPDH grew faster than VK-2-3, whereas RS.KdpC and RS.KdpC–GAPDH grew at the same rate as VK-2-3 (Figure 2; Supplementary Table S2). The residual sugar concentration in RS.KdpC, VK-2-3, and RS.KdpC–GAPDH fermentation broths were not significantly different; however, that in the fermentation broth of RS.GAPDH was significantly lower than that in the other four strains (Figure 3; Supplementary Table S2). The titers and yields of CoQ10 for RS.KdpC, VK-2-3, and RS.KdpC–GAPDH were not significantly different. Compared with VK-2-3, RS.GAPDH CoQ10 titer increased by 14%, NADH/NAD^+^ increased by 1.3-fold, ATP content increased two-fold, and CoQ10 yield increased by 8.6% (Figures 6A,B; Supplementary Table S2). RT-qPCR results revealed that the relative expression of *gapdh* and *kdpc* was 2.3-and 2.2-fold higher, respectively, in RS.GAPDH than VK-2-3. In contrast, the differences in the relative expression of *gapdh* in VK-2-3, RS.KdpC and RS.KdpC–GAPDH were not significant (Figure 5B; Supplementary Table S2). Li et al. reported that decreasing the NADH/NAD^+^ ratio in *E. coli* DH5α cells affects succinate synthesis. *E. coli* YL104 was metabolically engineered to use NADH dehydrogenase in all phases of succinate synthesis, thereby lowering the NADH/NAD^+^ ratio. Succinate yield increased by 7% in all phases of succinate synthesis in the engineered strain (Li, 2017). Koo et al. found that the intracellular NADH/NAD^+^ ratio was positively correlated with CoQ10 content in *A. tumefaciens* A603-35, a strain with high yield of CoQ10. GAPDH expression in A603-35 increased the NADH/NAD^+^ ratio from 0.8 to 1.2 at 48 h, and CoQ10 yield in the corresponding shake-flask increased from 2.16 mg/g to 3.63 mg/g (Koo et al., 2010). Zhu et al. overexpressed GAPDH and inhibited carotenoid synthesis in *R. sphaeroides* to increase CoQ10 production to 63.7 mg/l, which was 27.8% higher than the control (Zhu, 2017). In this study, RS.GAPDH showed the greatest growth compared with other strains, indicating that the overexpression of *gapdh* accelerated electron transfer in the respiratory chain, which subsequently increased CoQ10 requirement, thus increasing bacterial concentration per unit volume per unit time. Moreover, overexpressing GAPDH increased NADH/NAD^+^. Driven by this reducing power, the metabolic process of the cell accelerated and glucose consumption increased, thus increasing CoQ10 production. Overexpressing KdpC alone reduced intracellular ATP concentration, but CoQ10 production did not increase, suggesting that KdpC might not regulate CoQ10 synthesis. The tandem overexpression of KdpC–GAPDH reduced intracellular ATP concentration and increased NADH/NAD^+^; however, CoQ10 titer did not increase, possibly because it affected the metabolic flow distribution of bacteria and increased metabolic pressure in cells, thus affecting CoQ10 synthesis (Perrakis and Romier, 2008). In addition, the sugar consumption of RS.GAPDH increased, indicating an increase in the ability of individual strains to use sugar. In contrast, the sugar concentration in the fermentation broth decreased, facilitating the treatment of three wastes and has social benefits.

### Results of the 5-L fermenter experiment

**Figure 7:**
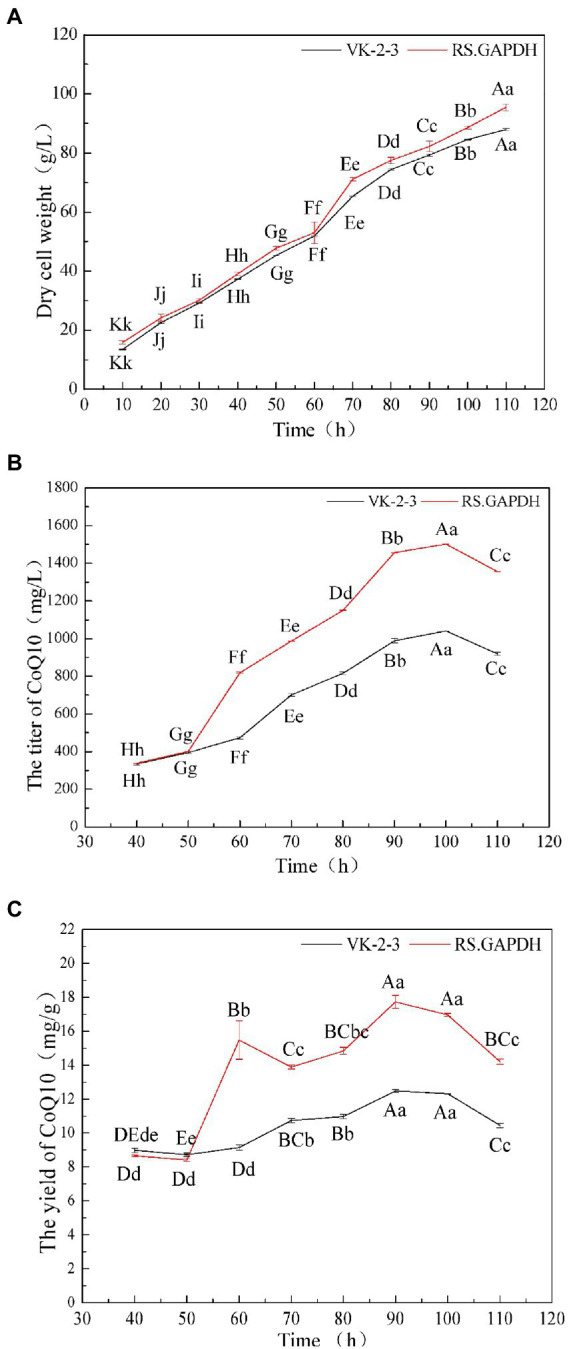
5-L fermenter experiment results. **(A)** The dry cell weights of the VK-2-3 and RS.GAPDH fermenters. **(B)** Coenzyme Q10 titer of VK-2-3 and RS.GAPDH **(C)** coenzyme Q10 yields of VK-2-3 and RS.GAPDH. Shoulder-notes in uppercase letters indicate highly significant differences (*p* < 0.01) and lowercase letters indicate significant differences (*p* < 0.05).

The dry cell weight of the bacteria gradually increased during fermentation. The dry cell weight of RS.GAPDH was 9% higher than that of VK-2-3 at the end of fermentation (Figure 7A; Supplementary Table S2). After 40–100 h, the CoQ10 titers of VK-2-3 and RS.GAPDH gradually increased with fermentation time. When the fermentation period reached 110 h, the CoQ10 titer declined. The CoQ10 yield of VK-2-3 was the maximum at 90 h, but the difference was not significant compared with the CoQ10 yield at 100 h. The CoQ10 yield of RS.GAPDH was the maximum at 90 h, but it was not significantly different from that at 100 h. Further, CoQ10 titers and yields of RS.GAPDH increased by 44.3 and 37.8%, respectively, compared with VK-2-3 after fermentation for 100 h (Figures 7B,C; Supplementary Table S2). Yang et al. used 15-L tanks to ferment and culture *R. sphaeroides* EIM-8, supplementing them intermittently with complete medium and continuously with glucose to extend culture time. CoQ10 yields and biomass values were 1289.76 mg/l and 40.92 g/l, respectively (Yang et al., 2013). Ren et al. increased CoQ10 production in *R. sphaeroides* to 129.97 mg/l by adding fermentation precursors in a 5-L fermenter, an increase of 42.9% compared with no addition of precursor (Ren, 2010). Han et al. cultured *R. sphaeroides* CF02-AF3-674 in a 2-L fermenter for 90 h and using HPLC, determined that the CoQ10 yield was 2037.4 μg/ml. The fermentation tank was scaled-up to verify the results. After 90 h of culture, the CoQ10 yield was 2693.5 μg/ml, which is 32% higher than that in the 2-L fermentation tank (Han et al., 2019). In this study, CoQ10 titer and yield of RS.GAPDH reached a value maximum after 100 h of batch fermentation and subsequently began decreasing; one possible reason for this could be that in RS.GAPDH, electron transfer in the respiratory chain gets accelerated under high-density culture conditions; bacterial growth and glucose consumption correspondingly increased. Therefore, cells need more CoQ10 to conduct metabolic processes. Simultaneously, because oxygen demand differs with the stages of fermentation, a controlled oxygen mode with regulated speed and ventilation is used in batch replenishment fermentation to optimize fermentation conditions. Low dissolved oxygen is used at the initial stages of cell growth to enable rapid plateauing, and higher dissolved oxygen levels during product synthesis help achieve remarkable results. CoQ10 and its structural analogs share some common pathways, and changing conditions of oxygen supply may affect its metabolic flux distribution; therefore, more precursor substances were used CoQ10 synthesis. CoQ10 production began to decline in the later stages of fermentation, indicating that cell density was extremely high, and some cells died from insufficient oxygen in the fermentation broth, thus rapidly reducing CoQ10 accumulation.

## Conclusion

In this study, we subjected the components of the glycolytic and energy conversion pathways to metabolic engineering and generated three strains with high yield of CoQ10, RS.PKAC, RS.PFK–PKAC, and RS.GAPDH. CoQ10 titers for RS.PKAC, RS.PFK–PKAC, and RS.GAPDH were 13, 6, and 14% higher and the yields were 16.7, 6.4, and 4% higher than those for VK-2-3, respectively. The sugar consumption and production performance of the three strains were higher than that for VK-2-3. The RT-qPCR results revealed that the relative expression of *pfk* and *pkac* genes in the RS.PKAC strain was 1.9 and 1.6 times higher than those in VK-2-3, respectively. However, the relative expression of *pkac* gene decreased by 47% in RS.PFK strain compared with that in VK-2-3. And the relative expression of *gapdh* and *kdpc* genes was 2.3-and 2.2-fold higher, respectively, in RS.GAPDH than VK-2-3. In contrast, the differences in the relative expression of *gapdh* gene in VK-2-3, RS.KdpC and RS.KdpC–GAPDH were not significant. Further, VK-2-3 and RS.GAPDH were cultured in a 5-L fermenter, and the results showed that the CoQ10 output and yield of RS.GAPDH increased by 44 and 38%, respectively. Our findings provide insights into the advantages of metabolic engineering for improving product yield and strain expansion.

## Data availability statement

The datasets presented in this study can be found in online repositories. The names of the repository/repositories and accession number(s) can be found in the article/Supplementary material.

## Author contributions

LZ performed the material preparation, data collection, and analysis, operated the experiment, analyzed the experimental data, and wrote the first draft of the manuscript. LZ and Z-yL designed the experiment. Y-lL and J-hH provided the analysis tools. All authors contributed to the article and approved the submitted version.

## Funding

This research was supported by the funds from the National Natural Science Foundation of China (32060017), Inner Mongolia Science and Technology plan project, Scientific and Technological Achievements Transformation project of Inner Mongolia (CGZH2018133), and Inner Mongolia Grassland Talent projects for team and individual.

## Conflict of interest

The authors declare that the research was conducted in the absence of any commercial or financial relationships that could be construed as a potential conflict of interest.

## Publisher’s note

All claims expressed in this article are solely those of the authors and do not necessarily represent those of their affiliated organizations, or those of the publisher, the editors and the reviewers. Any product that may be evaluated in this article, or claim that may be made by its manufacturer, is not guaranteed or endorsed by the publisher.

## Footnotes

^1^ http://www.genome.jp/kegg/annotation/

## Abbreviations

PFK: phosphofructokinase
PKAC: cyclic AMP-dependent protein kinase
KdpC: adenosine triphosphate hydrolase
GAPDH: glyceraldehyde-3-phosphate dehydrogenase
VK-2-3: Rhodobacter sphaeroides VK-2-3
V-0: Rhodobacter sphaeroides V-0
HPLC: High performance liquid chromatography
L. lactis: Lactococcus lactis
SAS: statistical analysis system
A603-35: Agrobacterium tumefaciens A603-35
dxs: 1-deoxy-D-xylulose-5-phosphate synthase
dxr: 1-deoxy-D-xylulose-5-phosphate reductoisomerase
HK: hexokinase
PK: pyruvate kinase
PC: pyruvate carboxylase
