# Supplementary material for: Overexpression of enzymes in glycolysis and energy metabolic pathways to enhance coenzyme Q10 production in Rhodobacter sphaeroides VK-2-3
Source: Front Microbiol. 2022 Aug 12;13:931470. doi: 10.3389/fmicb.2022.931470 (PMC9412181; doi:10.3389/fmicb.2022.931470)
Supplement: Supplementary file 1 [file Data_Sheet_1.docx]

Supplementary Material

# 1 Transformation of Bacteria with Recombinant Plasmid

*Escherichia coli* DH5α competent cells were transformed with the recombinant plasmid using the heat-shock method because of its high conversion rate. Then, plasmids were extracted from *E. coli* DH5α and transferred into *R. sphaeroides* VK-2-3 competent cells prepared in our laboratory. Electrotransformation was used because the heat shock method had a low transformation rate for these competent cells. The heat-shock method was used following the manufacturer’s instructions. Electrotransformation was performed as follows:

1. Electrode cups were soaked in 75% alcohol for 15 min, washed with anhydrous ethanol for 10 min, washed two times with ddH_2_O, dried, and placed in an ice bath.
2. A total of 1 µL of recombinant plasmid was added to 100 µL of *R. sphaeroides* VK-2-3 competent cells (without mixing). The mixture was added to the grooves of the two electrode pads in the electrode cup and placed in an ice bath for 30 min.
3. The electrode cup was placed in the instrument, and the voltage was adjusted to 0.8, 1, 1.2, 1.4, and 1.8 kV. Electric shock was given for 5.4–5.8 ms. The electrode cup was immediately removed and 800 μL of LB medium was added to the cup. Then, the bacteria were cultured with shaking at 32°C and 220 rpm for 2 h.
4. A total of 50 μL of the sample was evenly spread onto media containing amp resistance (100 μg/mL) and cultured upside down at 32°C for 5–6 d.

The *R. sphaeroides* VK-2-3 competent cells were prepared as follows:

1. The *R. sphaeroides* VK-2-3 bacterial solution was diluted and spread onto media A for single-colony cultivation. Single colonies with good growth were picked using bamboo sticks and cultured in 100 mL of media B overnight at 32°C and 220 rpm.
2. The bacterial solution was inoculated at 1% and transferred to 50 mL of media B. The culture was shaken at 32°C and 220 rpm for about 1 h to OD_600_ = 0.58–0.7 and placed in an ice bath for 10 min. The bacterial solution was separated into two pre-cooled 50-mL centrifuge tubes.
3. The solution was centrifuged at 4°C and 4500 rpm/min for 10 min, and the supernatant was discarded. Fifteen milliliters of pre-cooled 0.1 mol/L CaCl_2_–MgCl_2_ solution was added to each tube and gently pipetted to resuspend the cells. The tubes were placed in an ice bath for 10 min.
4. The solution was centrifuged at 4°C and 4500 rpm/min for 10 min, and the supernatant was discarded. One milliliter of precooled 0.1 mol/L CaCl_2_–MgCl_2_ solution was added to each tube and gently pipetted to resuspend the cells. The tubes were placed in an ice bath for 10 min.
5. Approximately 0.25 mL of pre-cooled sterile glycerin was added to each tube, placed in an ice bath for 10 min, and distributed into twenty 1.5-mL sterile centrifuge tubes. Then, 100 μL of glycerin was dispensed into each tube. The tubes were stored at −70°C until use.

# 2 Results of Recombinant Strain Construction

# 2.1 Target gene amplification and double-enzyme digestion recovery results

The results of target gene amplification and double-enzyme digestion recovery are shown in Figure S1.

## 2.2 Verification of double-enzyme digestion of the recombinant plasmid

The results of double-enzyme digestion of the recombinant plasmid are shown in Figure S2.

## 2.3 Results of recombinant plasmid sequencing

The sequencing results are shown as follows:

**Sequence 1**: *pfk*

1 ATGATCCCCA TTCTCACGTT GACGCTGAAC CCGGCGATCG ACCTCGCGGC CGACGTGCCG

61 CAGGTCCTGC CGGGCATCAA GCTGCGCTGC ACCGAGCCCC GGGTCGATCC GGGCGGCGGC

121 GGTCTGAACG TCAGCCGCGC CATCCGCATC CTCGGCGGCC GGAGCACCGC CTTCGTGGCC

181 CTGGGCGGCA ACATCGGCGG GCGGCTGGCC GCCCTGGTCG CGGCGGCCGG CATCGAGATC

241 GTGCCCTTCT CGGGCCCCGG CGAGACGCGC GAAAGCCTCA CCGTCACCGA AACGGCCACG

301 GGCCGGCAGT TCCGCTTCAT GCTGCCGGGC GCCGCCTGGG ACGCAGAGCG CGTCGAGGCC

361 GCTCTGGCCC GGATCGACCG CGCCGTGCCC GAAGGCGGCA TGGTGGTCCT CTCGGGGTCG

421 CTGCCGCCCG GCGTGCCGGC CGACTTCCCG GCCATGGTCT CCCGGGTGCT GGGCAAGCGC

481 GCCCGGCTTC TGGTCGACAC GTCCGGCGCG CCGCTCGCCC ATCTCGCCGC GGGCGGGGTG

541 CCCGACCTCG ACATCCTGCG GATGGACGAT GGCGAGGCCG CGAGCCTCGC CGGCCGCCCC

601 CTCGCCTGCG CCTCCGAAAC GGCCGATTTC GCCTCGATCC TCGTGGCCCG CGGCGTGGCC

661 GAGTGCGTGA TCGTCGCCCG CGGCGCCGAC GGCTCGGTCC TCGCCGATGC CCGCGGCCGC

721 TGGCACGCCC GCTCCGAACC GGTCGAGGTG GTGAGCGCGG TGGGTGCGGG CGACACGTTC

781 GTCGGCGCCT TCGTCCTCGC CCTCTCGCGG GGCGCCCCGC CGGAAGAGGC CCTGGCCCAT

841 GGCGTGGCCG GCGCCGCGGC GGCCGTCCTC ACCGAGGCGA CCGAACTCTG CCATCCCGAG

901 GATGTGGCGC GCCTCCTGCC CTCCTGTGCC GCGACGGCCC TCTGA

**Sequence 2**: *pkac*

1 ATGTTCATAC ACGATCCTGA CCCGACAATC ACCGATTGCA GAAACTGTCC GCTCCGGCGG

61 AAACCGCTGT TCCTTCCCTT CTCCGACAGC GAGCTCTCCT TCATGGAGCA GTTCAAGGTG

121 GGCGAGCTGG TCGTCGCGCC CGGCGTCACT GTGCTCGAGG AGGGGCAGGG CAGCGCGCAT

181 CTCTTCACCG TCCTGAGCGG GCTCGGCATC CGCTCGACCA TGCTCGAGAA CGGCCGGCGT

241 CAGGTCATCA ACTTCCTCTT CCCGGGCGAT TTCATCGGGC TGCAGGCCGG TCTGGCGGGA

301 GAGATGCGCC ATTCGGTGGA AAGCACGACC ACCATGGTGC TCTGTGTCTT CAACCGCGCG

361 GATCTGTGGG ATCTGTTCCG GGAAGAGCCG GAGCGTGCCT ACGACCTCAC CTGGATCGCA

421 GCGGTCGAGG AGCATTTCCT GGGCGAGACC ATCGCCTCGC TCGGCCAGCG GGACGCGACC

481 GAGCGGCTGG CCTGGGCGCT GCTGCGCATC CATGAGCGGC TGTCGGCCAT CGGCCTCGCC

541 GAGCGGGGCC GGGTGCCGAT GCCCTGGCGG CAGCAGGATC TGGCGGATGC GCTGGGACTG

601 TCGCTCGTTC ACACCAACAA GACGATCCGC CGCCTGCGCG AGACGGGCCA CGCGCTGTGG

661 GAGGGGGGCA CCCTGTTCGT CGACCGGGAG CGGCTCGCCA CGCTGGCACT GGCCGATCCC

721 GACCGTCCGC GCCGCAGGCC CCTCATCTGA

**Sequence 3**: *kdpc*

1 ATGATGACCC ATCTCCGCCC CGCGCTGGCG AGCCTTCTGG CGCTGAGCCT GCTGACCGGC

61 GTGGCCTATC CGCTGGCCCT GACCGGCCTC GCGGCCGTCA TCGCCCCCGA CCGCGCCGCG

121 GGCAGCCTGA TCCTGCGCGA GGGGCAGGTC GTGGGCTCGG CCCTGATCGG GCAGGGCTTC

181 GAGGGCCCGG GCTATCTGCA TCCCCGTCCC TCGGCGAGCG ACTGGAACGC GGCCGGCACC

241 TCCGCCTCGA ACCTCGGGCC GACCTCGGCT GCGCTGCTGG CCCAAGTGCA GGAGCGGCAG

301 ACGGCCTATG AGGCGCAAAA CGGCGCCTCC GCTCCGGTCG ATGCGGTCAC CGCCTCGGGC

361 AGCGGGCTCG ATCCCCATGT CTCGCCCGCC AATGCCCGGG CGCAGGCGGG CCGCATCGCC

421 CGCGCCCGCG GCCTGGAGGA GGCCGCCGTG CGCCGCCTGA TCGAGGCCCA TGTCGAGCCG

481 CCGCTGCTGG GTCTCTGGGG GCAGGCGCGG GTCAATGTGC TGGCCGTCAA CCTCGCGCTC

541 GACGCGGCCG GGGCCTGA

**Sequence 4**: *gapdh*

1 ATGACCGTGA AAGTGGCAAT CAACGGCTTC GGCCGCATCG GGCGGAACGT GCTCCGCGCC

61 ATCATCGAAT CGGGCCGGAC CGATATCGAG GTGGTGGCGA TCAACGATCT CGGCCCGGTC

121 GAGACCAACG CGCACCTGCT GCGCTTCGAC TCGGTCCACG GCCGCTTCCC CGCCACCGTC

181 ACCACCACCG AGAAGACCAT CGACGTGGGC CGCGGCCCGA TGGATGTGAC CGCGATCCGC

241 AACCCGGCCG AGCTTCCCTG GGGCCATGTC GACATCGTGA TGGAATGCAC CGGCATCTTC

301 ACCGACAAGG AGAAGGCGAA GATCCACCTC GAGAACGGCG CCAAGCGCGT GCTGGTCTCC

361 GCCCCCTCGA CCGGCGCGGA CAAGACCATC GTCTTCGGCG TGAACCACGA GACGCTGACG

421 AAGGACGATC TCGTCGTCTC GAACGCCTCC TGCACGACGA ACTGCCTCTC GCCGGTGGCC

481 AAGGTGCTGA ACGACACGAT CGGCATCACC AAGGGCTTCA TGACCACGAT CCACAGCTAC

541 ACCGGCGACC AGCCGACGCT GGACACAATG CACAAGGATC TCTACCGCGC GCGGGCCGCG

601 GCGCTGAGCA TGATCCCCAC CTCGACCGGC GCCGCCAAGG CCGTGGGCCT CGTGCTGCCG

661 GAACTGAAGG GCAAGCTCGA CGGCGTGGCG ATCCGGGTGC CGACGCCGAA CGTCTCGGTG

721 GTGGACCTCG TGTTCGAAGC CTCGCGCGCG ACCAGCGTCG AGGAAGTGAA CGCCGCCATC

781 CGCGAGGCCG CCGACGGCAA GCTGAAGGGC ATCCTCGGCT ATACCGACCA GCCCAACGTC

841 TCGATGGACT TCAACCACGA TCCGCACAGC TCGATCTTCC ACCTCGACCA GACCAAGGTC

901 ATGGAAGGCA ACATGGTGCG GATCCTGACC TGGTACGACA ACGAATGGGG CTTCTCGAAC

961 CGCATGGCCG ATACGGCCGT GGCCATGGGC AAGCTCATCT GA

The sequencing result confirmed the target gene sequence. Thus, the recombinant plasmid was constructed successfully. The recombinant strains were labeled RS.PFK, RS.PKAC, RS.PFK-PKAC, RS.KdpC, RS.GAPDH, and RS.KdpC-GAPDH.

# 3 Detection and Quantitative Analysis of NAD^+^, NADH, and ATP

1. Drawing of the standard curve: 0.1 g of NADH, NAD^+^, and ATP were dissolved in 1 L of 0.2 mol/L phosphate-buffered saline (pH = 7.0) to prepare 100 mg/L stock solutions, which were diluted to different concentrations (Table S6). High-performance liquid chromatography (HPLC) was performed at 254 nm and standard curves were drawn.
2. Mobile phase preparation for the NAD^+^ and NADH assay: 10.93 g of NaH_2_PO_4_ and 3.04 g of Na_2_HPO_4_ were dissolved in water. Then, 3.22 g of tetrabutylammonium bromide was added to the solution. The pH of the solution was adjusted to 6.5 with 10% NaOH. The solution was filtered under vacuum and volume-adjusted to 1 L. This was mixed with acetonitrile at a ratio of 86:14 (v:v).
3. Mobile phase preparation for the ATP assay: 13.6 g of KH_2_PO_4_ was added to 152 mL of 0.1 mol/L NaOH. The volume was adjusted to 1 L and the pH to 6.5.
4. Absorption was detected at 254 nm using a C_18_ column (4.6 mm × 150 mm × 5 µm). The flow rate was 0.8 mL/min, and the column temperature was 35°C.
5. Standard curves for NAD^+^, NADH, and ATP were drawn (Figure S3).

**Table** S**1.** Primer sequences of the target gene

| Gene name | Primer sequences |
| --- | --- |
| *pfk*-F | 5′-AAGGATCCATGATCCCCATTCTCACGTT-3′ |
| *pfk*-R | 5′-ATAAAGCTTTCAGAGGGCCGTCGCG-3′ |
| *pkac-*F | 5′-CCCAAGCTTATGTTCATACACGATCCTGACC-3′ |
| *pkac-*R | 5′-ATTGGTACCTCAGATGAGGGGCCTGC-3′ |
| *gapdh-*F | 5′-CCAAGCTTATGACCGTGAAAGTGGCAATC-3′ |
| *gapdh-*R | 5′-CCGGTACCTCAGATGAGCTTGCCCAT-3′ |
| *kdpc-*F | 5′-ACCGGATCCATGATGACCCATCTCC-3′ |
| *kdpc-*R | 5′-ATATATAAGCTTTCAGGCCCCGGCCGC-3′ |
| RT-*rpoZ*-F | 5′-TTCGAGCTGGTGATGCT-3′ |
| RT-*rpoZ*-R | 5′-ACTCGATCTGGGTCTGG-3′ |
| RT-*pfk*-F | 5′-TCACGTTGACGCTGAACCC-3′ |
| RT-*pfk*-R | 5′-CCGTTTCGGTGACGGTGAG-3′ |
| RT-*pkac*-F | 5′-ACCATGGTGCTCTGTGTCTT-3′ |
| RT-*pkac*-R | 5′-CGATCCAGGTGAGGTCGTAG-3′ |
| RT-*gapdh-*F | 5′-GGACCGATATCGAGGTGGTG-3′ |
| RT-*gapdh-*R | 5′-GTCGATGGTCTTCTCGGTGG-3′ |
| RT-*kdpc-*F | 5′-GGCTCGATCCCCATGTCTC-3′ |
| RT-*kdpc-*R | 5′-GTTGACGGCCAGCACATTGA-3′ |

Note: The underline represents the restriction site: GGATCC-*Bam*HI, AAGCTT-*Hind*III, and GGTACC-*Kpn*I

**Table S2.** Raw Data

Note: Double click on the form to open the original form.

**Table S3.** Standard curve determination

| 100 mg/L of stock solution (mL) | PBS (mL) | Dilution (mg/L) |
| --- | --- | --- |
| 0 | 10 | 0 |
| 0.5 | 9.5 | 5 |
| 1.0 | 9.0 | 10 |
| 1.5 | 8.5 | 15 |
| 2.0 | 8.0 | 20 |
| 2.5 | 7.5 | 25 |
| 3.0 | 7.0 | 30 |
| 3.5 | 6.5 | 35 |
| 4.0 | 6.0 | 40 |
| 4.5 | 5.5 | 45 |

PBS: phosphate-buffered saline

**Table S4.** Strain and plasmid information

| Type | Name | Source |
| --- | --- | --- |
| Strains | *Rhodobacter sphaeroides* V-0 | Provided by Shenzhou Biological Co., Ltd., Hohhot, Inner Mongolia, China. It is primarily used for experimental research. |
|  | *Rhodobacter sphaeroides* VK-2-3 | *Rhodobacter sphaeroides* VK-2-3 (VK-2-3) was obtained by compound mutagenesis of the original strain of *Rhodobacter sphaeroides* V-0 using heavy ions and high voltage prick electric fields. Its CCTCC NO is M 2021735. |
|  | RS.PFK | They were obtained by genetic modification of VK-2-3. |
|  | RS.PKAC |  |
|  | RS.PFK-PKAC |  |
|  | RS.KdpC |  |
|  | RS.GAPDH |  |
|  | RS.KdpC-GAPDH |  |
|  | *Escherichia coli* DH5α | Purchased from Beijing Tiangen Biological Co., Ltd., China. |
| Plasmid | pBBR1MCS-4 | It was gifted by Mr. Li Feng from Tianjin University, China. It has ampicillin resistance, and it is a broad host plasmid vector that can be stably expressed. |

| 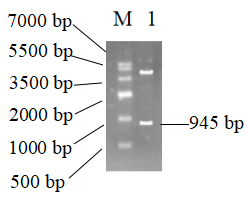 | 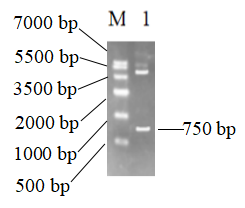 | 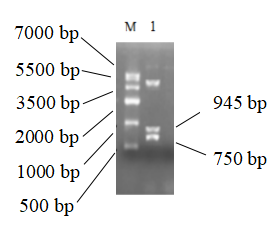 |
| --- | --- | --- |
| (A) | (B) | (C) |
| 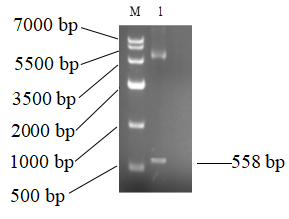 | 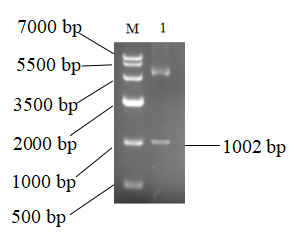 | 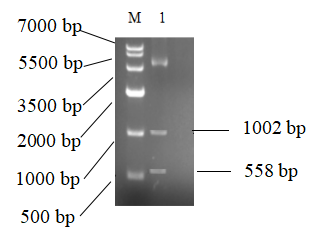 |
| (D) | (E) | (F) |
| **Figure S1.** Verifying double-enzyme digestion results of the recombinant plasmids. (A) *pfk*, (B) *pkac*, (C) *pfk-pkac*, (D) *kdpc*, (E) *gapdh*, and (F) *kdpc-gapdh*. | | |

|  |
| --- |
| A |
|  |
| B |
|  |
| C |
| **Figure S2.** Standard curves. (A) NAD^+^, (B) NADH, and (C) ATP. Concentrations of NAD^+^, NADH, and ATP are plotted on the *X*-axes. Peak areas of HPLC chromatograms (at 254 nm) are plotted on the *Y*-axes. |
